# Care for low back pain: can health systems deliver?

**DOI:** 10.2471/BLT.18.226050

**Published:** 2019-04-30

**Authors:** Adrian C Traeger, Rachelle Buchbinder, Adam G Elshaug, Peter R Croft, Chris G Maher

**Affiliations:** a Institute for Musculoskeletal Health, University of Sydney, PO Box M179, Missenden Road, Camperdown NSW 2050, Australia.; b Department of Epidemiology and Preventive Medicine, Monash University, Melbourne, Australia.; c Menzies Centre for Health Policy, University of Sydney, Sydney, Australia.; d Institute of Primary and Health Care Sciences, Keele University, Newcastle, England.

## Abstract

Low back pain is the leading cause of years lived with disability globally. In 2018, an international working group called on the World Health Organization to increase attention on the burden of low back pain and the need to avoid excessively medical solutions. Indeed, major international clinical guidelines now recognize that many people with low back pain require little or no formal treatment. Where treatment is required the recommended approach is to discourage use of pain medication, steroid injections and spinal surgery, and instead promote physical and psychological therapies. Many health systems are not designed to support this approach. In this paper we discuss why care for low back pain that is concordant with guidelines requires system-wide changes. We detail the key challenges of low back pain care within health systems. These include the financial interests of pharmaceutical and other companies; outdated payment systems that favour medical care over patients’ self-management; and deep-rooted medical traditions and beliefs about care for back pain among physicians and the public. We give international examples of promising solutions and policies and practices for health systems facing an increasing burden of ineffective care for low back pain. We suggest policies that, by shifting resources from unnecessary care to guideline-concordant care for low back pain, could be cost-neutral and have widespread impact. Small adjustments to health policy will not work in isolation, however. Workplace systems, legal frameworks, personal beliefs, politics and the overall societal context in which we experience health, will also need to change.

## Résumé

Les lombalgies sont la principale cause d'années de vie vécues avec une incapacité dans le monde. En 2018, un groupe de travail international a invité l'Organisation mondiale de la Santé à attirer l'attention sur la charge que représentent les lombalgies et sur la nécessité d'éviter le recours excessif aux solutions médicales. En effet, selon les dernières recommandations cliniques internationales, de nombreux cas de lombalgie ne nécessitent pas ou peu de traitement formel. Lorsqu'un traitement est requis, il est recommandé de limiter la prise d'analgésiques, les injections de stéroïdes et la chirurgie rachidienne, et d'encourager plutôt les thérapeutiques physiques et psychologiques. Très souvent, les systèmes de santé ne sont pas conçus pour appliquer cette approche. Dans cet article, nous abordons les raisons pour lesquelles un changement des systèmes s'impose si l'on veut prendre en charge les lombalgies suivant les recommandations. Nous détaillons les principales difficultés de la prise en charge des lombalgies dans le cadre des systèmes de santé. Il s'agit notamment des intérêts financiers des laboratoires pharmaceutiques, entre autres; des systèmes de paiement obsolètes qui privilégient la prise en charge médicale à l'autogestion par les patients; et de croyances et traditions médicales profondément ancrées parmi les médecins et la population. Nous donnons des exemples internationaux de solutions, de politiques et de pratiques prometteuses pour les systèmes de santé confrontés de plus en plus souvent à une prise en charge inefficace des lombalgies. Nous suggérons des politiques qui, sans incidence sur les coûts, en transférant les ressources allouées aux soins inutiles vers des soins conformes aux recommandations, pourraient avoir un impact considérable. De petits ajustements des politiques de santé ne suffiront cependant pas. Les systèmes des milieux professionnels, les cadres juridiques, les croyances personnelles, les politiques et le contexte sociétal global dans lequel s'inscrit la santé devront également changer.

## Resumen

El dolor lumbar es la causa principal de vivir con discapacidad durante años en todo el mundo. En 2018, un grupo de trabajo internacional pidió a la Organización Mundial de la Salud que prestara más atención a la carga del dolor lumbar y a la necesidad de evitar soluciones excesivamente médicas. De hecho, las principales directrices clínicas internacionales reconocen ahora que muchas personas con dolor lumbar requieren poco o ningún tratamiento formal. Cuando se requiere tratamiento, el enfoque recomendado es desalentar el uso de analgésicos, inyecciones de esteroides y cirugía de la columna vertebral y, en su lugar, promover las terapias físicas y psicológicas. Muchos sistemas de salud no están diseñados para apoyar este enfoque. En este documento, se expone por qué el cuidado del dolor lumbar de acuerdo con las directrices requiere cambios en todo el sistema. Se detallan los retos clave de la atención del dolor lumbar en los sistemas de salud. Estos incluyen los intereses financieros de las compañías farmacéuticas y de otro tipo, los sistemas de pago obsoletos que favorecen la atención médica por encima del autocuidado de los pacientes, así como las tradiciones y las creencias médicas profundamente arraigadas sobre la atención del dolor de espalda entre los médicos y el público general. Se presentan ejemplos internacionales de soluciones prometedoras y de políticas y prácticas para los sistemas de salud que se enfrentan a una carga cada vez mayor de la atención ineficaz para el dolor lumbar. Se sugieren políticas que, al desplazar los recursos de la atención innecesaria a la atención acorde con las directrices para el dolor lumbar, podrían ser neutras en cuanto a costes y tener un impacto generalizado. Sin embargo, los pequeños ajustes en la política sanitaria no funcionarán de forma aislada. Los sistemas del lugar de trabajo, los marcos jurídicos, las creencias personales, la política y el contexto social general en el que vivimos la salud también tendrán que cambiar.

## ملخص

آلام أسفل الظهر هي السبب الرئيسي على مستوى العالم لقضاء سنوات من الحياة في ظل الإعاقة. في عام 2018، طالبت مجموعة عمل دولية منظمة الصحة العالمية بزيادة الاهتمام بأعباء آلام أسفل الظهر، والحاجة إلى تجنب المحاليل الطبية بشكل مفرط. والواقع أن المبادئ التوجيهية السريرية الدولية الرئيسية الحالية تدرك أن العديد من الأشخاص الذين يعانون من آلام أسفل الظهر يحتاجون إلى القليل من العلاج الرسمي، أو لا يحتاجون إليه. عندما يكون العلاج مطلوبًا، فإن الأسلوب الموصى به هو الابتعاد عن استخدام دواء للألم، وحقن الستيرويد، وجراحة العمود الفقري. وبدلا من ذلك يتم الترويج للعلاجات البدنية والنفسية. إلا أن العديد من النظم الصحية ليست مصممة لدعم هذا الأسلوب. نناقش في هذا البحث لماذا تتطلب رعاية آلام أسفل الظهر المتوافقة مع الإرشادات، تغييرات في النظام بالكامل. نحن نتناول بالتفصيل التحديات الرئيسية للعناية بآلام أسفل الظهر في النظم الصحية. وهي تشمل المصالح المالية لشركات الأدوية وغيرها من الشركات؛ مثل أنظمة الدفع القديمة التي تفضل الرعاية الطبية على الإدارة الذاتية للمرضى؛ والتقاليد والمعتقدات الطبية الراسخة بشأن رعاية آلام الظهر بين الأطباء والعامة. نحن نعطي أمثلة دولية للحلول والسياسات والممارسات الواعدة للنظم الصحية التي تواجه عبئاً متزايدا من الرعاية غير الفعالة لآلام أسفل الظهر. كما نقترح سياسات يمكنها - من خلال تحويل الموارد من الرعاية غير الضرورية إلى الرعاية المتوافقة مع المبادئ التوجيهية لآلام أسفل الظهر - أن تكون محايدة من حيث التكلفة ولها تأثير واسع الانتشار. إلا أن عمليات الضبط البسيطة في السياسة الصحية لن تعمل بمفردها. حيث سيحتاج أيضاً للتغيير كل من أنظمة مكان العمل، وأطر العمل القانونية، والمعتقدات الشخصية، والسياسات، والطابع المجتمعي العام الذي نواجهه في الصحة.

## 摘要

腰痛是与全球残疾人士相伴数年的一项主要病因。2018 年，某国际工作小组呼吁世卫组织加强对腰痛负担的关注，并尽力避免过度医疗。事实上，主要国际临床指南现已认可腰痛患者只需极少治疗或无需正式治疗。使用止痛药、注射类固醇和进行腰椎手术并非值得鼓励的推荐疗法，相应地，患者应改善生理和心理状态。但许多卫生系统并非为支持此疗法设计。在本文中，我们讨论了符合指南的腰痛护理为何需要进行系统性变革，并详述了卫生系统中腰痛护理的关键挑战，包括制药公司与其它公司的经济利益；过时的医疗保险偿付系统更倾向于让患者接受医疗服务而非自我管理；以及医生与公众对腰背痛护理的医疗传统和信仰早已根深蒂固。我们为因腰痛护理无效而面临沉重负担的卫生系统提供了一些国际案例，包含颇有前景的解决方案、政策和实践。我们建议施行成本适中、有广泛影响的政策，将不必要的护理资源转换成符合指南的腰痛护理。然而，卫生政策方面的微小调整无法单独起作用，这同时也需要工作场所制度、法律框架、个人信仰、政治以及我们所期望的卫生系统整体社会环境的相应改变。

## Резюме

Боль в поясничной области является основной причиной многолетней инвалидности во всем мире. В 2018 году международная рабочая группа призвала Всемирную организацию здравоохранения уделять больше внимания бремени боли в поясничной области и необходимости исключения чрезмерного медицинского вмешательства. В связи с этим основные международные клинические рекомендации на настоящий момент признают, что большинству пациентов с болями в поясничной области практически не требуется формального лечения. В случае если лечение необходимо, рекомендуемый подход не советует использовать обезболивающие препараты, инъекции стероидов и операции на позвоночнике, предлагая вместо этого активно использовать физическую терапию и психотерапию. Многие системы здравоохранения не в состоянии поддерживать данный подход. В этой статье авторы обсуждают причины, по которым рекомендуемая медицинская помощь при боли в поясничной области требует реформирования всей системы здравоохранения. Авторы подробно описывают основные проблемы лечения боли в поясничной области в рамках различных систем здравоохранения. Среди них следует отметить финансовые интересы фармацевтических и других компаний; устаревшие платежные системы, делающие упор на оказание медицинской помощи вместо обучения пациента методам самопомощи; глубоко укоренившиеся традиции и убеждения у врачей и широкой общественности относительно медицинской помощи при боли в поясничной области. Авторы приводят примеры возможных международных решений, а также политики и практические подходы для систем здравоохранения, сталкивающихся с растущим бременем неэффективной помощи при боли в поясничной области. В статье предлагаются процедуры и методики, которые могут не требовать дополнительных расходов и иметь широкомасштабное воздействие благодаря переключению ресурсов с оказания ненужной медицинской помощи на согласующуюся с рекомендациями помощь при боли в поясничной области. Однако небольшие корректировки политики в области здравоохранения не будут эффективными, если они будут проводиться изолированно друг от друга. Необходимо также преобразование систем на рабочих местах, правовых основ, личных убеждений, политик и общественного контекста, связанных со здравоохранением.

## Introduction

Low back pain is the single biggest cause of years lived with disability worldwide, and a major challenge to international health systems.^1^ In 2018, the *Lancet* Low Back Pain Series Working Group identified a global problem of mismanagement of low back pain.^2^^–^^4^ The group documented the phenomenon of unnecessary care in both high- and low-income settings, whereby patients receive health services, which are discordant with international guidelines.^2^^–^^4^ The articles summarized the strong evidence that unnecessary care, including complex pain medications, spinal imaging tests, spinal injections, hospitalization and surgical procedures, is hazardous for most patients with low back pain.^2^^–^^4^

Although we could not find systematic estimates for the worldwide prevalence of unnecessary care for low back pain, the CareTrack studies provide some indication of scale. Those studies estimated that 28% (95% confidence interval, CI: 19.7–38.6) of health care for low back pain in Australia (based on 164 patients receiving 6488 care processes)^5^ and 32% (95% CI: 29.5–33.6) of health care for low back pain in the United States of America (based on 489 patients receiving 4950 care processes)^6^ was discordant with clinical guidelines. The figures are likely an underestimate because they did not include diagnostic imaging tests. The upward trend in unnecessary care for low back pain is even more concerning. One meta-analysis from 2018 found that simple imaging tests were requested in one quarter of back pain consultations (415 579 of 1 675 720 consultations) and the rates of complex imaging (e.g. magnetic resonance imaging) had increased over 21 years.^7^ There is no robust evidence of benefit for spinal fusion surgery compared with non-surgical care for people with low back pain associated with spinal degeneration.^8^ However, over the years 2004–2015, elective spinal fusion surgery in the United States increased by 62.3% (from 60.4 per 100 000 to 79.8 per 100 000), with hospital costs for this procedure exceeding 10 billion United States dollars (US$) in 2015.^9^ In 2014, 3–4% of the adult United States population (9.6 million to 11.5 million people of 318.6 million) were prescribed long-term opioid drug therapy, in many cases because of chronic low back pain.^10^ The *Lancet* working group called on the World Health Organization to increase attention on the burden of low back pain and “the need to avoid excessively medical solutions.”^4^

The movement away from medicalized management of low back pain is reflected in recent clinical guidelines. All six of the major international clinical guidelines released since 2016 prioritized non-medical approaches for patients with low back pain (Box 1).^11^^–^^16^ Primary-care clinicians following these guidelines would manage uncomplicated cases with advice, education and reassurance. For patients at risk of developing chronic pain and disability, clinicians would, depending on which guidelines they followed, consider offering treatments such as spinal manipulation, massage, acupuncture, yoga, mindfulness, psychological therapies or multidisciplinary rehabilitation. Most health systems are not well-equipped to support this approach.

Box 1 **Key messages from six international clinical guidelines for management of low back pain**

- Adopt a stepped or stratified approach to care of low back pain, guided by the patient’s response to previous care or the results of risk prediction tools. Recommended by 4 out of 6 guidelines.^11^^–^^14^
- First step care for low back pain, which will be sufficient for many patients, is to provide advice to remain active, education on the benign nature of low back pain and reassurance about the absence of serious pathology. Recommended by all guidelines.^11^^–^^16^
- Second step options for acute low back pain include physical therapies (massage, spinal manipulation, heat-wrap therapy), psychological therapies (psychologically informed physiotherapy) or complementary therapies (acupuncture^a^). At least one recommended by all guidelines.^11^^–^^16^
- Second step options for chronic low back pain comprise physical therapies (exercise, massage, spinal manipulation), psychological therapies (cognitive behavioural therapy), complementary therapies (mindfulness-based stress reduction, yoga, acupuncture,^a^ tai chi). Recommended by 4 out of 6 guidelines.^11^^–^^13^^,^^15^
- Third step in chronic low back pain care is multidisciplinary pain management (targets physical, psychological and social aspects of low back pain and involves a team of clinicians). Recommended by 5 out of 6 guidelines.^11^^–^^15^
- Care of low back pain care without medication is preferred. Recommended by all guidelines.^11^^–^^16^
- If pain medication is needed, begin with a nonsteroidal anti-inflammatory drug at the lowest effective dose for the shortest time. Recommended by all guidelines.^11^^–^^16^
- Avoid prescribing opioid drugs for low back pain where possible. Recommended by 3 out of 6 guidelines.^11^^,^^14^^,^^16^
- Do not offer injectable steroid drugs to patients with chronic non-specific low back pain. Recommended by 3 out of 6 guidelines.^11^^,^^13^^,^^14^
- Do not offer surgery for patients with non-specific low back pain outside of a randomized trial. Recommended by 3 out of 6 guidelines.^11^^,^^13^^,^^14^

^a^ Acupuncture was endorsed by the United States, Danish and Australian guidelines, but discouraged by United Kingdom and German guidelines. Belgian guidelines made no recommendation.Notes: We analysed current clinical guidelines on low back pain care from six countries (United States of America, United Kingdom of Great Britain and Northern Ireland, Australia, Germany, Belgium and Denmark) released since 2016. Some specific details of recommendations differed between the guidelines.

Discontinuing unnecessary care of low back pain is beneficial for patients. We argue that safer therapies should be offered, even though the evidence base for their effectiveness is not yet clear enough to achieve consistent endorsement across guidelines.^11^^–^^16^ In this paper we expand on the policy challenge of ensuring care for low back pain is concordant with guidelines and we outline potential solutions for health systems.

## Health-system challenges

### Access to suitable therapies

While most people with low back pain will require little or no formal care, for those who do require extra help an immediate challenge is patients’ and clinicians’ lack of access to the recommended therapies (Box 1). For example, a German survey found that general practitioners fundamentally agreed with the content of clinical guidelines for low back pain, but almost half had no access to the recommended multidisciplinary approach to pain management.^17^ A more recent qualitative study of general practitioners in the United Kingdom of Great Britain and Norther Ireland concluded the same; one general practitioner viewed recommendations to provide a course of non-pharmacological care as “lovely pie-in-the-sky plans.”^18^

People living in rural and remote areas are often unable to access multidisciplinary pain management because it is typically provided in tertiary health-care settings in cities. For example, a person with chronic low back pain living in Kununurra in rural Western Australia, which has a population of 5600, would have to travel 827 km to Darwin or 3040 km to Perth to access their nearest multidisciplinary pain management service.^19^ Patients may also have limited access to recommended physical and psychological therapies, and complementary therapies such as tai chi and yoga.

Bringing together necessary health services for people with complex chronic conditions is a growing challenge for modern health systems. Australia’s chronic disease management programme was designed to coordinate services across multiple providers. The programme, however, permits too few visits for proper delivery of effective coordinated care for chronic low back pain, since it only allows for a total of five visits per annum per patient, shared across all allied health services and irrespective of the number of chronic conditions a patient has. For chronic low back pain, a programme of primary care-based cognitive behavioural therapy can consist of seven sessions^20^ and a programme of mindfulness-based stress reduction can be eight sessions.^21^ Exercise programmes could similarly exceed this cap; an effective yoga programme was shown to comprise 12 sessions.^22^ Some patients would also have to allocate one or more of their five sessions to manage comorbidities, such as diabetes or obesity. The costs of providing this care are far lower than some of the unnecessary care options. For example, the reimbursement offered by Medicare, Australia’s publicly funded health insurance scheme, for the five visits is only 311 Australian dollars (AU$), whereas spinal fusion surgery in a New South Wales public hospital is covered up to a cost of AU$ 53 700.^23^

### Lack of time and training

The new guidelines require longer, more complex consultations. General practitioners cite time pressure and lack of confidence in new approaches to care as barriers to adherence to guidelines.^24^ Providing prognosis-specific care (Box 1) in a 5-minute consultation^25^ is next to impossible when treating a patient with severe pain, a poor prognosis or chronic low back pain with multiple comorbidities. One survey of 6588 consultations for low back pain in Australia found that only around one fifth (21 of 100) of general practitioners performed a complete history and physical examination.^26^

Most of the recommended second-step treatment options require referral to clinicians with specific training. Some health systems, especially those in low- and middle-income countries, will not have the supply of clinicians to deliver these therapies at scale. For example, Nepal has only one physiotherapist per 20 000 people, compared with 24 per 20 000 in Australia.^27^

Furthermore, even when patients can access multidisciplinary pain management services, they may not receive care that is concordant with guidelines.^28^ A patient living with chronic low back pain in West Virginia, a state with the highest opioid drug overdose rate in the United States, could receive a service that offers opioid medication, but not evidence-based care such as pain education, coping strategies or rehabilitation services.^29^

### Funding arrangements

Numerous companies and individuals profit from health care for low back pain. For example, Purdue Pharma L.P., makers of the opioid painkiller OxyContin^®^ (oxycodone), has an estimated worth of US$ 13 billion. Oxycodone is the most used drug for chronic non-cancer pain in Australia.^30^ In the United Kingdom, prescriptions for opioids including oxycodone are increasing, despite evidence of poor efficacy and substantial harm to patients.^31^ In 2013, prescription opioids were responsible for 44 000 drug overdose deaths in the United States.^10^ A systematic review of randomized trials found that opioids were of limited benefit in chronic low back pain, even in dangerously high doses,^32^ and a recent trial found opioids led to slightly worse pain outcomes than non-opioid medication for back pain and osteoarthritis.^33^ The current international low back pain guidelines provide mixed messages regarding opioids. However, the Centers for Disease Control and Prevention guideline for prescribing opioid drugs for chronic pain are clear; extended-release opioids such as oxycodone should never be used for initial management of chronic non-cancer pain.^34^

Fines for breaking the regulations around marketing of medicines to doctors may not deter wealthy companies who want to increase their market share. Even a multi-million-dollar settlement would be tiny for a major pharmaceutical company that markets pain medicines. OxyContin^®^ is estimated to have generated approximately US$ 35 billion in revenue for its manufacturer.^35^ The largest settlement that we are aware of was around US$ 2.3 billion in 2009 paid by Pfizer Inc. to settle a misleading marketing case concerning Lyrica^®^ (pregabalin), a commonly prescribed drug for low back pain. The fine represented less than 5% of the company’s US$ 50 billion revenues that year. Regulators should keep in mind that such large companies can accept any fine as a cost of doing business in the area of pain relief.

Health-service funding arrangements too can discourage the less-is-more approach that suits most cases of uncomplicated, non-specific low back pain. Fee-for-service health systems, for example, tend to provide incentives for activity and volume of care and hence inadvertently lead to more unnecessary care. A Cochrane review of systems of payment compared health service measures in two randomized controlled trials and two controlled before–after studies of 640 primary-care physicians and 6400 patients. The review found that fee-for-service systems had higher numbers of contacts, visits to specialists and diagnostic and curative services compared with capitation systems.^36^ Capitation systems, particularly those where clinicians receive a fixed salary to provide care for those enrolled in a given location, can reduce the number of services provided. However, capitation funding may also have undesirable effects, such as encouraging clinicians to provide the most time-efficient rather than the most effective care. Systems designed to solve these issues, such as pay-for-performance systems and quality-based contingency payments, may not reward clinicians fairly for all the complexities involved in treating people with low back pain.

While knowledge of best practice for low back pain has evolved, our health systems and their funding mechanisms have not kept up. As a result, many health-care systems internationally continue to fund guideline-discordant care, such as opioid drugs, radiofrequency denervation and spinal fusion surgery (Box 1). These treatments were common practice before a robust system of assessment of best practice was in place. Current funding arrangements therefore present a challenging evidence–policy–practice paradox. For example, Australian Medicare, and many private health insurers, do not fund guideline-concordant self-care by patients, such as yoga and tai chi, and provide limited funding for supervised exercise programmes. There are two interrelated issues here. First, some of these alternative services lack a sufficiently robust evidence base that would allow them to pass an assessment, which is rightly, a prerequisite for being added to the Australian Medicare fee-for-service schedule. Second, Medicare does not have a record of funding such alternative, non-medical interventions, although this could change if the evidence base for these services were to improve and a submission was made for their inclusion in the schedule of payments.

## Achieving guideline concordance

**Fig. 1:**
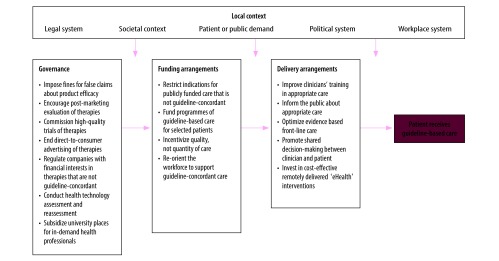
Health system levers to increase concordance with guidelines for care of low back pain

Notes: The boxes are arranged, from left to right, in order of health policy level: governance refers to the highest policy level, followed by funding arrangements, then delivery arrangements. The arrows indicate that policies at one level can have downstream effects on other levels, ultimately influencing the likelihood that a patient receives guideline-based care. Some policies in this example will be easier to implement than others, depending on local context. Local context factors that are external to, but interact with, components of the health system, are likely to influence delivery of guideline-concordant care.

Some of the above challenges may appear insurmountable. However, we believe that with coordinated efforts targeting each level of the health system, change is possible. In Table 1 we provide suggestions for delivery arrangements, financial arrangements and governance of international health systems to better support guideline-concordant care for low back pain. Although most examples of initiatives in Table 1 are taken from high-income countries, we believe many could be trialled in low- and middle-countries. Fig. 1 depicts a systems-level approach to achieving guideline concordance.

**Table 1:** Health-system barriers to following guideline recommendations on care for low back pain, and potential policy solutions

Guideline recommendation	Health-system barrier	Details	Potential policy solutions (suitability for health systems^a^)
Conduct a focused history and physical examination to determine patients’ risk of having a serious underlying cause of pain	Lack of time and training	Clinicians may lack adequate training in musculoskeletal assessment and management	Delivery arrangements • Increase training in history and examination procedures for low back pain and include the topics of unnecessary care and shared decision-making in curricula for trainee clinicians (all health systems) • Provide easy access to training courses for clinicianson shared decision-making for low back pain care (all health systems)^37^ • Provide locally relevant care pathways for low back pain e.g. the National Low Back and Radicular Pain Pathway 2017 in the United Kingdom (fee-for-service systems, capitation systems)^38^ • Build audit and feedback mechanisms on low back pain care, e.g. feedback on referral rates for diagnostic imaging tests (all health systems)^39^
		Clinicians may be under time pressure during consultations for low back pain	Delivery arrangements • Enhance the role of nurse practitioners and physiotherapists in primary care as they may be less likely to prescribe unnecessary care for low back pain, e.g. imaging tests (all health systems)^40^ • Assess the cost–effectiveness of using allied health staff who could provide equivalent low back pain care as physicians (all health systems)^41^ • Encourage evaluations, embedded in routine care, of the cost–effectiveness of any new model of low back pain care (all health systems) • Allow patients to self-refer to physical and psychological therapies care for low back pain (capitation systems) Financial arrangements • Provide reimbursement for clinicians needing extra time for patients with complex low back pain problems (fee-for-service systems, hybrid systems)
Screen patients using a prognostic model; arrange early referral to non-pharmacological treatment for those at risk of a poor outcome	Vested interests and funding arrangements	Some clinicians, companies and professional associations market ineffective early interventions for low back pain	Governance • Impose fines for clinicians, companies and professional associations who make false claims about efficacy of services (all health systems) Prohibit direct-to-consumer advertising of non-evidence-based tests and treatments (all health systems)
	Limited access to evidence-based information and health care	The prognosis of low back pain and the role of self-care is poorly understood by the public	Delivery arrangements • Create mass-media campaigns informing the public about self-management of low back pain, when to seek health care and how to identify false treatment claims (all health systems)^42^ • Create health literacy programmes about low back pain, e.g. school and podcast programmes targeting parents and their school-aged children (all health systems)^43^ • Encourage shared decision-making between clinician and patient on low back pain care, which can also increase informed decision-making for other health conditions
Prioritize non-pharmacological treatment for initial management	Limited access to coordinated, evidence-based health care	Physical, psychological and complementary therapies for low back pain may be unaffordable for patients	Delivery arrangements • Invest in existing eHealth programmes for low back pain care, e.g. clinician-guided, remotely delivered, cognitive behavioural therapy-based pain management programmes (all health systems)^44^
		Evidence-based non-pharmacological treatment for low back pain is poorly integrated with general practitioner care.	Financial arrangements • Fund programmes of guideline-adherent non-pharmacological treatment for selected patients with low back pain, e.g. those at risk of chronic pain (all health systems) • Limit or remove expensive, non-evidence-based treatments for low back pain from funding schedules (all health systems) Set up bundled payment systems for low back pain care, e.g. comprehensive care for joint replacement programme which “coordinates care over the full continuum of services and eliminates spending that doesn't benefit patients” (all health systems^45^
	Lack of time and training	Quality cognitive-behavioural therapy for low back pain is hampered by shortages of health workers, e.g. clinical psychologists	Governance • Provide government subsidies for university training positions on for low back pain care in needed health professions (all health systems)
If medication is needed, begin with simple analgesics such as nonsteroidal anti-inflammatory drugs	Vested interests and funding arrangements	Complex medicines for low back pain that lack evidence of lack of efficacy are aggressively marketed	*Governance* • Impose fines for pharmaceutical companies who make false claims about efficacy and safety of products (all health systems) • Require post-marketing evaluation to measure impact of use of medicine outside of the indications where efficacy has been demonstrated (all health systems)
		Medicines and procedures that are ineffective for low back pain are funded by public or private insurance schemes	Financial arrangements • Tighten or restrict indications for financial coverage of low back pain care, e.g. only fund treatment when there is evidence for clear benefit or in the context of a randomized trial to gather evidence (fee-for-service systems, capitation systems) Governance • Ensure agreements between all stakeholders involved in funding, provision and evaluation of therapies for low back pain and that evaluations are only done in the context of a clinical trial (all health systems) • All clinical trials for low back pain treatment should pre-specify what outcomes constitute positive and negative results (all health systems) • Require regular health technology assessments and reassessments of health services for low back pain^46^ (all health systems)
		Over-the-counter medicines that are either ineffective (paracetamol) or untested (codeine combinations) in low back pain are cheap and easy to access from community pharmacies	Governance • Change opioid drugs, e.g. codeine combinations, from over-the-counter to prescription-only medicine (all health systems)
Avoid the following: (i) prescribing opioid drugs; (ii) referral for routine diagnostic imaging tests; (iii) prescribing steroid injections for patients with chronic low back pain; and (iv) referral for surgery for patients with chronic non-specific low back pain, outside of a randomized trial	Vested interests and funding arrangements	Providers (physicians, radiologists and surgeons), device manufacturers and pharmaceutical companies profit from low back pain care	Governance • Compulsory review of all new drugs, equipment and practices for low back pain care, e.g. standard health technology assessment and reassessment (all health systems)^46^
	Limited access to coordinated, evidence-based health care	Patients, clinicians and the public believe that that opioid drugs, imaging tests and surgery are necessary care for low back pain	Delivery arrangements • Create mass-media campaigns to warn health providers and the public about unnecessary care for low back pain (all health systems)
	Vested interests and funding arrangements	Public or private insurance schemes reimburse patients for low back pain care that is not concordant with guidelines, e.g. opioid drugs, imaging tests and surgery	Financial arrangements • Tighten or remove indications for health-care coverage, e.g. only fund treatments for low back pain where there is evidence for clear benefit or, if there is absence of evidence, in the context of a randomized trial (fee-for-service systems, capitation systems)

^a^ We indicate which types of health-care funding systems are most suitable for interventions: all health systems (including low- and middle-income countries); fee-for-service systems (e.g. Australia); capitation systems (e.g. United Kingdom of Great Britain and Northern Ireland); hybrid systems, i.e. combination of fee-for-service and capitation (e.g. United Sates of America).

### Change ideas about back pain

Misconceptions about management of low back pain remain common. For example, around half of patients presenting with low back pain (144 of 300) believed diagnostic imaging tests were necessary.^47^ Targeting misconceptions at the population level through mass-media campaigns is an effective, but costly, approach. Campaigns such as the 1997–1999 campaign in Victoria, Australia, which encouraged people to “not take back pain lying down,” can change beliefs about low back pain and alter people’s behaviour, including the proportion of patients returning to work.^42^ The growth of social media should make similar campaigns easier and cheaper to implement. Targeting young people through health information messages on social media or other channels before unhelpful beliefs become entrenched is another worthwhile approach. The Informed Health Choices initiative in Uganda, which helped primary-school children and their parents detect false treatment claims, is evidence that these programmes can succeed.^43^ Whole-population education initiatives, such as these could be expanded to include information about unnecessary diagnostic tests and to target condition-specific health myths. However, those manufacturers or individuals who stand to gain financially from sales of certain therapies and who market opposing messages are a powerful force. Efforts to counteract such vested interests are likely to need sustained and coordinated support from the legislative, labour, health and government sectors (Fig. 1).

Clinicians require more training and educational support from health systems if they are to use new approaches to back pain care. Educational materials^48^ and workshops^49^ can improve care quality. Key topics could include emphasizing the need for a history and physical examination in patients with low back pain and building skills in addressing patient concerns and requests for unnecessary care, such as imaging tests in the absence of clinical features of serious pathology. Decision-making shared with the patient can reduce unnecessary tests in other non-serious pain conditions,^50^ although it remains unclear exactly how health systems can improve uptake of shared decision-making between clinicians and patient.^51^

A more immediate solution would be to borrow behavioural approaches that have shown promise in other areas of health care. Behavioural interventions, for example, can counteract cognitive biases and improve clinical decision-making. Something as simple as a letter to clinicians, noting their poor prescribing habits in comparison with their peers, can have a substantial impact. A recent randomized trial by the Australian health department involved sending peer-comparison letters to 6649 high-prescribers of antibiotics. The outcome was a reduction in their prescriptions for inappropriate antibiotics from 109.3 to 95.8 scripts per 1000 consultations (12.3% reduction over 6 months).^52^ A similar trial found sending peer comparison letters to 5055 high-prescribers of antipsychotic drugs reduced prescriptions from 2864 to 2456 patient days on quetiapine per prescriber (adjusted difference, −319 days of 2864; 11.1% fewer days over 9 months).^53^

Another strategy to increase delivery of guideline-concordant care could be redesigning electronic health records. An observational study conducted in two emergency departments in Pennsylvania in the United States found that making 10 tablets the default option for prescriptions in the system was associated with a 22.8% increase in prescriptions for 10 tablets (from 20.6% to 43.3% of 3264 prescriptions) and a 6.7% decrease in prescriptions for 20 tablets (from 22.8% to 16.1% of 3264 prescriptions) over 4 weeks.^54^ Electronic health record systems are increasingly being used to collect clinical data, auto-populate risk prediction tools with relevant clinical and demographic data, and default to the most appropriate strategy for that person’s risk profile. While trials of such innovations are needed to determine their optimal design and assess their acceptability and usefulness, such approaches have the advantage that they could be implemented on a large scale and at relatively low cost.

### Incentivize high-value care

Aligning funding models with best-practice care for low back pain could be difficult. As mentioned above, funding schedules already cover many non-evidence based items which remain popular with clinicians and patients and are difficult to remove funding from.^55^ One example of success was the removal of the vertebroplasty procedure from the Australian Medicare funding schedule. A key factor in this achievement, however, was that vertebroplasty had only interim funding status that was contingent on trial results. When the trials did not show positive outcomes, funding for the therapy was denied, albeit with the controversy that comes with restricting or removing access. Funding is unlikely to shift from established care practices unless there is clear evidence for the superior safety, effectiveness and cost–effectiveness of the alternatives. Funding decisions are complex and rarely are such decisions influenced solely by evidence. Lobbying from vested interests, media coverage and communication around funding decisions, patient and clinician resistance to changes, as well as the current political climate, and the alignment of these factors with public opinion, also play an influential role (Fig. 1). The public needs to have a better understanding of the shortcomings of some types of established medical care. Many patients may already have such understanding (Box 2) and, in fact, patients could help drive changes to the system by lobbying for care that is evidence-based (see Local context, Fig. 1). In Oregon in the United States, patients, clinicians and policy-makers recently designed a new way to pay for appropriate care for low back pain. Oregon will be the first American state to reallocate Medicaid funds away from ineffective and potential harmful therapies for low back pain, such as long-term opioids, to evidence-based, non-medical treatments.^56^

Box 2 **Examples of patient perspectives on management of chronic low back pain**

**Example 1**

A patient with many years of chronic arthritis and back pain:

“What I want and have always wanted is to stay positive, keep the pain at a comfortable level, and stay independent. Big things that have helped me were a good rapport with my boss so I could work some days from home and have my desk and seat adapted….joining in with groups for regular exercise to keep me mobile (aqua aerobics for me!)…and feeling valued so I am seen as ‘me the worker or the volunteer’ and not ‘me as the person with the pain’.

Medical treatments, including strong painkillers, have certainly helped at particular times of my life. But I really wish that, years ago, when all the pain began, there had been messages like the ones in the guidelines now. I wish there had been someone suggesting things to try for myself and to be positive about staying active and learning ways of getting on with life despite the pain. Doctors shouldn’t be nervous about suggesting people try things like heat packs and exercise first before reaching for tablets or injections – it could be a real ‘lightbulb’ moment for the patient.

But I know just how difficult this is when doctors are so busy. I was proud to be involved in a scheme for patients to help other patients with advice about simple things like public transport when they were anxious about even trying it. I have gained such a lot from doing things for myself, and I like the idea of recommending and funding more help and support for other patients with back pain to learn how to do the same – and shifting from care being all about drugs and injections.”

**Example 2**

A former nurse who has had back pain for many years:

“I started with back pain when I was 30 [years old] and it became so bad when I was nursing that I went from paracetamol to codeine to morphine patches. I had read about the patches and insisted my doctor prescribed them even though he was not too keen. The patches did help the pain, but they made me feel worse and I gave up after a few months. I had to give up nursing because I couldn’t move patients and that upset me. And then I decided to tackle the back pain myself.

A physiotherapist gave me some exercise sheets – 15 years later I still have them and use them. I lost weight, I stopped heavy lifting. The pain is there but I can cope with it. Not only did I get my pain under control, I felt so much better in myself. I now gauge my activity by what I feel my body can manage – [it’s] so much better than popping pills even though I still take the occasional painkiller to help when the back pain is bad. I get help from talking with other people. That’s what people need when they get back pain – someone who has time to listen, understands the pain, and helps them to find ways to stay active and engaged by way of exercise and work, rather than just giving a prescription for painkillers.”

Source: The two patients are members of the patient and public involvement panel at Keele University’s Institute of Primary and Health Care Sciences, England. They provided these thoughts after reading a draft of the paper before submission.

The optimal way to pay clinicians who treat low back pain remains unclear. Modest financial incentives for providing guideline-concordant care are unlikely to change practice.^57^ There is, however, potential for episode-of-care reimbursement or risk-adjusted capitation payment models to provide incentives for complying with guidelines. Simulation models using data from 969 medical practices in the United States found that replacing fee-for-service with fixed monthly capitation payments, to cover all costs associated with providing primary care, could encourage the delivery of more care outside of consultations without financial losses from the government health-care budget.^58^

### Regulate vested interests

Governments will have a key role to play in supporting new approaches to managing low back pain. Some shifts have already begun. The Australian Government scheduled codeine products as prescription-only drugs in February 2018. This change is likely to reduce the overuse of these medicines, although the broader consequences of such policies remain unclear. In 2010, in the United States, after government reduced access to a formulation of OxyContin^®^ that was easily abused, use of the drug dropped substantially (from 35.6% to 12.8% of 2566 patients), but many patients who abused both formulations (66 of 100) simply switched to using heroin.^59^ Any attempt to restrict public access to opioids should therefore be accompanied by adequate access to addiction services, social programmes and evidence-based non-pharmacological alternatives, as well as programmes to accurately monitor use of opioids.

Changes to governance arrangements will have to occur not just in health systems, but also in the complex framework in which health systems operate. Encouraging a shift away from unnecessary medical care requires support from governments, workplaces, legislative systems, consumers and professional bodies (Fig. 1).

## Conclusion

Delivery of guideline-concordant care for low back pain requires system-wide changes. Strong governance at each level of the health system will be key to redefining how society views and manages low back pain. Health systems should prioritize policies that: empower clinicians and consumers to make well-informed choices; encourage clinicians to deliver the right care to those who need it most; provide financial support to evidence-based non-pharmacological treatment; and regulate the influence of those with vested interests in the current situation. Small adjustments to health policy will not work in isolation. Workplace systems, legal frameworks, personal beliefs, politics and the overall societal context in which we experience health, will also need to change. Addressing system-level barriers to guideline-based care could be cost-neutral; every year health systems waste billions of dollars on unnecessary tests and treatments for low back pain. Although disinvestment is difficult, redistributing funds to support guideline-concordant care is a promising way forward. Because current approaches to treatment often lack formal evidence, we strongly encourage careful evaluation of any new approach to funding or service delivery.
